# *Paecilomyces lilacinus* Vaginitis in an Immunocompetent Patient

**DOI:** 10.3201/eid0909.020654

**Published:** 2003-09

**Authors:** Jeanne Carey, Ron D’Amico, Deanna A. Sutton, Michael G. Rinaldi

**Affiliations:** * Beth Israel Medical Center, New York, New York, USA; † University of Texas Health Science Center at San Antonio, San Antonio, Texas, USA; ‡ Audie L. Murphy Division, South Texas Veterans Health Care System, San Antonio, Texas, USA

**Keywords:** *Paecilomyces*, vaginitis

## Abstract

*Paecilomyces lilacinus,* an environmental mold found in soil and vegetation, rarely causes human infection. We report the first case of *P. lilacinus* isolated from a vaginal culture in a patient with vaginitis.

*Paecilomyces lilacinus*, a saprobic filamentous fungus, found in soil, decaying vegetation, saunas, and laboratories (as an airborne contaminant), is an infrequent cause of human disease (*1*,*2*). Most cases of disease caused by the genus *Paecilomyces* occur in patients who have compromised immune systems, indwelling foreign devices, or intraocular lens implants (*2*,*3*). Rarely has disease been reported in immunocompetent hosts without any identifiable risk factor.

We describe the first case of *P. lilacinus* isolated from a vaginal culture in a patient with vaginitis and review the published literature addressing *P. lilacinus* infections in immunocompetent patients. Our review demonstrates that the reports of *P.* *lilacinus* infections in immunocompetent hosts have become more frequent in the last several years. This trend indicates that *P. lilacinus* may be an emerging pathogen.

## Case Report

A 48-year-old woman reported vaginal itching and discharge of 5 months’ duration. Her symptoms had been recalcitrant to several courses of therapy for a presumptive diagnosis of candidal vaginitis. She had been treated initially with fluconazole, then sequentially with topical clotrimazole, ticoconazole ointment, and intravaginal boric acid gel. Her medical history was notable for mild gastritis (treated with omeprazole) and irregular uterine bleeding, controlled with hormone replacement therapy (a transdermal estrogen/progesterone combination). The patient was in a monogamous relationship with her husband but reported abstinence for several months because of the severity of her vaginal symptoms.

On physical examination, vaginal erythema with a white liquid vaginal discharge was observed. Although a potassium hydroxide (KOH) preparation was not obtained at baseline, the discharge grew *P. lilacinus* in pure culture.

The patient was treated empirically with itraconazole, 200 mg orally twice a day for 3 weeks. At the end of therapy, she reported complete resolution of her vaginal discharge and a significant decrease in her vaginal pruritus. A repeat vaginal culture was not obtained at her first follow-up appointment after completion of itraconazole therapy because the vaginal vault contained a large amount of blood. At an appointment 6 months later, she remained free of vaginal discharge; a vaginal fungal culture and KOH preparation performed at that time were negative.

The results of laboratory studies, including serum protein electrophoresis (with immunoglobulin [Ig] G, IgA, IgM) C3, C4, erythrocyte sedimentation rate, a complete blood count, CD4 cell count, and CD8 cell count were all within normal limits. Results of a test for antibodies to HIV were negative. An anergy panel (with *Candida* and *Trichophytin* used as controls) was reactive. A purified protein derivative was not placed because the patient had a history of a positive test result.

The patient’s isolate was forwarded to the Fungus Testing Laboratory, Department of Pathology, University of Texas Health Science Center at San Antonio, Texas, for confirmation of the identity and antifungal susceptibility testing, and accessioned into the stock collection as UTHSC 01-872. The isolate was initially subcultured onto potato flakes agar (PFA, prepared in house), which was prepared in-house, at 25°C, 30°C, 35°C, and 40°C (ambient air with alternating daylight and darkness). The isolate was subsequently plated onto carnation leaf agar (CLA [prepared in-house]) and malt agar (Remel, Lenexa, KS) at 25°C. Temperature studies were repeated after initial observations.

The case isolate was evaluated for susceptibility to antifungal agents by using the National Committee for Clinical Laboratory Standards broth macrodilution method M38-P (*4*). Briefly, the case isolate and the *P. variotii* control organism, UTHSC 90-450, were grown on PFA for 7 to 10 days at 25°C to induce conidial formation. The mature PFA isolate and control slants were overlaid with sterile distilled water, and suspensions were made by gently scraping the colonies with the tip of a Pasteur pipette. Heavy hyphal fragments were allowed to settle, and the upper, homogeneous conidial suspensions were removed. Conidia were counted with a hemacytometer, and the inoculum was standardized to 1.0 x 10^5^ CFU/mL. Conidial suspensions were further diluted 1:10 in medium for a final inoculum concentration of 1.0 x 10^4^ CFU/mL. Final drug concentrations were 0.03–16 μg/mL for amphotericin B (Bristol-Myers Squibb, Princeton, NJ)**,** ketoconazole (Janssen Pharmaceutica, Titusville, NJ) and clotrimazole (Schering-Plough, Kenilworth, NJ), 0.125–64 μg/mL for 5-flucytosine (Roche Laboratories, Nutley, NJ), fluconazole, voriconazole (Pfizer, Inc, New York, NY), and terconazole (Ortho-McNeil Pharmaceuticals, Inc., Raritan, NJ), and 0.015–8 μg/mL for itraconazole (Janssen Pharmaceutica) and posaconazole (Schering-Plough).

## Results

**Figure 1:**
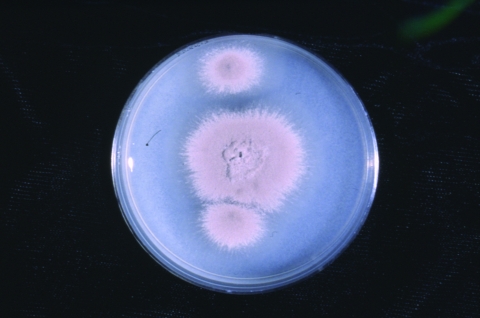
Macroscopic structure of case isolate after 2 weeks’ incubation at 25°C on potato flakes agar, prepared in house.

**Figure 2:**
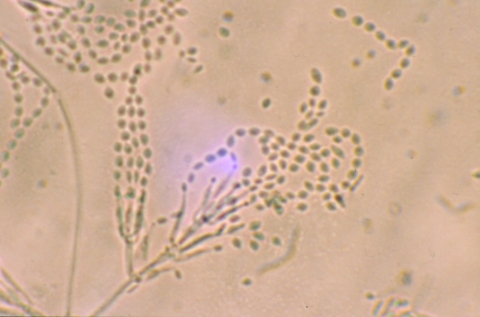
Divergent phialides and long, tangled chains of elliptical conidia borne from more complex fruiting structures characteristic of *Paecilomyces lilacinus*, 460X.

Growth of the isolate on PFA produced a buff-colored to slightly lavender, somewhat granular colony after 7 days’ incubation at 25°C. Repeat subcultures with extended incubation (up to 2 weeks) yielded colonies which were more definitely mauve-colored, consistent with those typically seen with *P. lilacinus* (Figure 1). The isolate failed to produce sporodochia on carnation leaf agar (CLA, prepared in-house), a feature seen with *Fusarium* species (many of which are lavender) and failed to produce a diffusing yellow pigment on malt agar, a characteristic seen with the closely related *P. marquandii.* The conidiogenous cells from the initial slide culture, held 7 days and prepared from a PFA block, consisted predominately of single, long, tapering phialides, somewhat atypical for the species. Repeat PFA slide cultures from subcultures displaying a more typical macroscopic structure yielded complex fruiting heads with verticillate conidiophores and divergent phialides, typical for *P. lilacinus* (Figure 2)*.* Conidiophore roughness, a feature described for *P. lilacinus*, was not observed, however, even after repeated subculturing and examinations. Smooth-walled, elliptical conidia occurred in long, tangled chains and measured approximately 2.0 X 2.5 μm. No chlamydospores were observed on any of the media examined. Temperature studies performed on two separate occasions (PFA) indicated 4+ growth at 25°C and 30°C, 2+ growth at 35°C, and no growth at 40°C.

Species of *Paecilomyces* known to produce pinkish to purplish colonies include *P. javanicus, P. fimetarius, P. fumosoroseus, P. lilacinus,* and *P. marquandii.* The first three species were excluded from consideration on the basis of the size of the conidia, as well as the lack of synnematal production for *P. fumosoroseus* (*5*). *P. marquandii* differs from *P. lilacinus* by the production of an intense yellow diffusible pigment, smooth-walled hyaline conidiophores, and the production of chlamydospores. Although the isolate in this case did display smooth conidiophores, no yellow pigment or chlamydospores were observed. The existence of intergrading forms between *P. lilacinus* and *P. marquandii* has been described (*6*). In such strains, characteristics of both species may be observed. On the basis of the characteristics above, the isolate was identified as *P. lilacinus*.

In vitro 48-hour to 72-hour MIC data in μm/mL for the isolate were as follows: amphotericin B, >16; 5-flucytosine (5-FC), >64; ketoconazole, 0.5/0.5; fluconazole, 32/64; itraconazole, 0.5/0.5; clotrimazole, 0.06/0.25; voriconazole, 0.25/0.25; terconazole, 4/8; and posaconazole, 0.125/0.125.

## Conclusions

*P. lilicanus* rarely causes human infection. A MedLine review of English-language literature from 1966 to 2003 yielded approximately 60 reports of *P. lilacinus* infections in patients who were immunocompromised, had undergone ophthalmologic surgery, or had indwelling foreign devices (*2*,*3*). A Medline review in the same time period indicated only six cases of *P. lilacinus* infections among patients who lacked a readily identifiable risk factor. A review of the bibliographies of relevant articles yielded three additional reports, for a total of nine cases in apparently immunocompetent hosts. The salient features of these cases, as well as ours, are summarized in the Table.

**Table:** Clinical features of cases of *Paecilomyces lilacinus* infections in immunocompetent hosts

Patient age, gender, and reference no.	Y	Type of infection	Treatment	Outcome
48-year-old woman (our case)	2002	Vaginitis	Iraconazole	Cure
36-year-old man (*3*)	1999	Cutaneous	Itraconazole	Cure
59-year-old woman (*7*)	2001	Cutaneous	Itraconazole	Cure
20-year-old woman (*8*)	1977	Cutaneous	Griseofulvin	Improvement, but not cure
19-year-old man (*9*)	1984	Cutaneous	Griseofulvin, then ketoconazole	Improvement
57-year-old man (*10*)	1999	Lung abscess	Surgery	Cure
20-year-old man (*11*)	1972	Pulmonary effusion	Amphotericin B	Cure
47-year-old woman (*12*)	1980	Sinusitis	Surgical debridement	Cure
34-year-old man (*13*)	1997	Endophthalmitis	Fluconazole, ketoconazole, itraconazole	Progression of disease
59-year-old woman (*14*)	1998	Onychomycosis	Terbinafine, various topical therapies, nail clipping	Progression of disease

The source of infection in most cases, including ours, is not easily identifiable. *P. lilacinus* has been isolated as a benign commensal organism on the toenails of immunocompetent hosts (*15*). In some cases, however, *P. lilacinus* has been pathogenic and has been implicated as a cause of onychomycosis in an immunocompetent adult (*14*). The low pathogenicity of this fungus in normal hosts is demonstrated by the indolent nature of two of the cutaneous infections listed in the Table (*8*,*9*), which were characterized by many years of chronic infection.

All isolates of the genus *Paecilomyces* should be tested for fungal susceptibility since clinical isolates of *P. lilacinus* frequently display considerable resistance. Isolates of *P. lilacinus*, for example, are usually resistant to amphotericin B and 5-flucytosine and susceptible to miconazole and ketoconazole, whereas isolates of the species *P. variotii* are usually susceptible to amphotericin B and 5-flucytosine (*16*). On the basis of breakpoints established for other fungi, the case isolate appeared resistant to amphotericin B, 5-flucytosine, fluconazole, and possibly terconazole. The approved azoles, itraconazole, ketoconazole, and clotrimazole, appeared susceptible, as did the investigational triazoles, voriconazole, and posaconazole.

Although the source isolate was susceptible to clotrimazole, the patient’s symptoms did not resolve after clotrimazole treatment. However, the duration of therapy with this agent and the degree of the patient’s adherence to the treatment regimen are unknown; one or both of these factors may have contributed to treatment failure.

Our review demonstrates that reports of *P. lilacinus* infections in immunocompetent hosts appear to be increasing. The four earliest cases occurred from 1972 to 1984, with one case reported every 3–5 years. The eight subsequent cases occurred between 1996 and 2002, for an average of slightly more than one new case per year.

We report the first case of *P. lilacinus* isolated from a vaginal culture in a patient with vaginitis, whose symptoms failed to improve after treatment with fluconazole. Her symptoms resolved after treatment with itraconazole, to which the case isolate was susceptible. *P.* *lilacinus* has been described as an emerging opportunistic pathogen in humans (*17*). In May 2002, the first case of disseminated *P. lilacinus* infection in an HIV-infected patient was reported (*18*). Our review suggests that *P. lilacinus* may be an emerging pathogen in immunocompetent adults as well.
